# Diffuse Pulmonary Lymphangiomatosis

**DOI:** 10.5334/jbsr.1603

**Published:** 2018-10-04

**Authors:** Caroline Ernotte, Laurent Médart, Laurent Collignon

**Affiliations:** 1 CHR Liège, BE

**Keywords:** lymphangiomatosis, thoracic malformation

## Case Report

**Figure 1:**
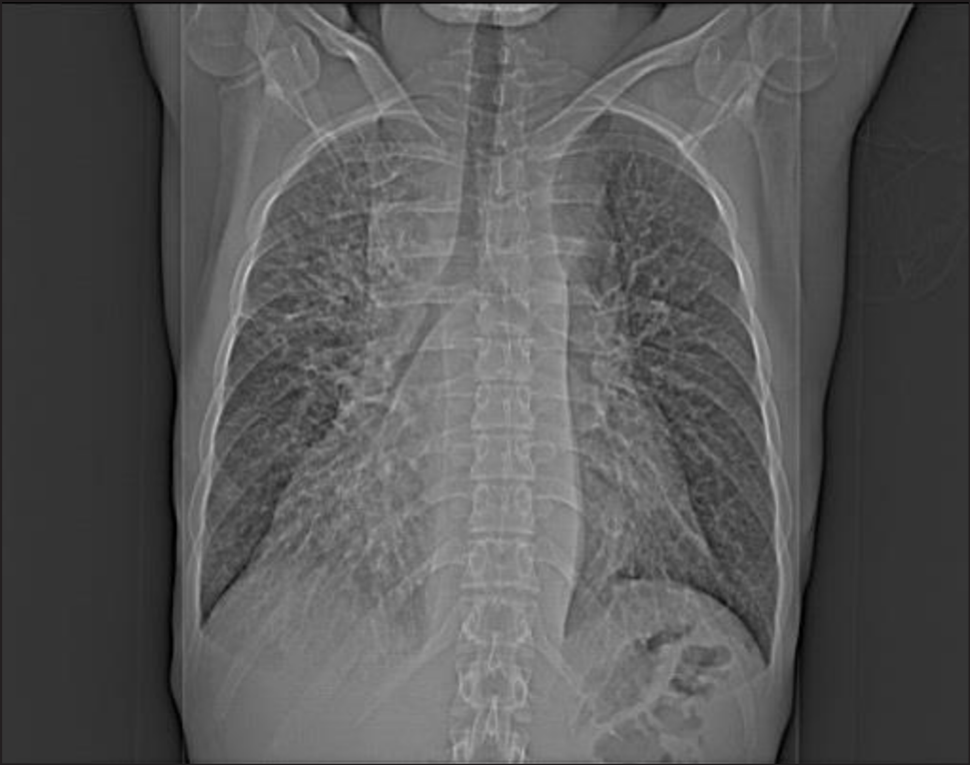


**Figure 2:**
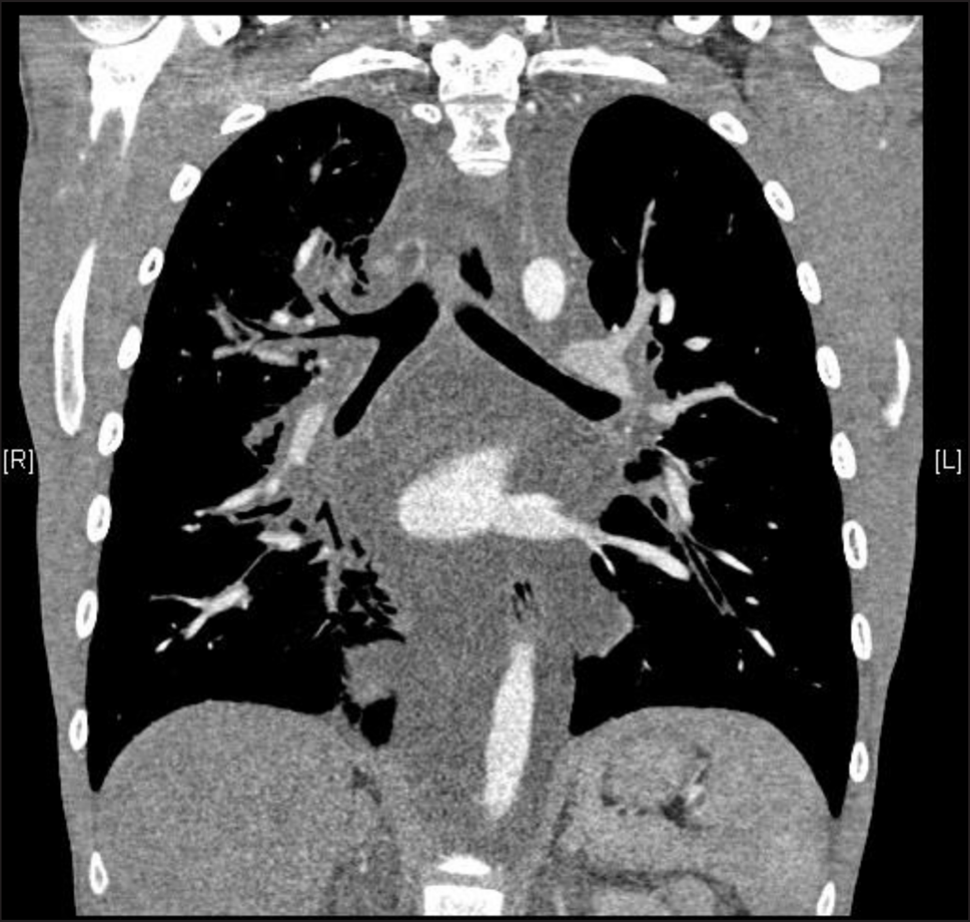


**Figure 3:**
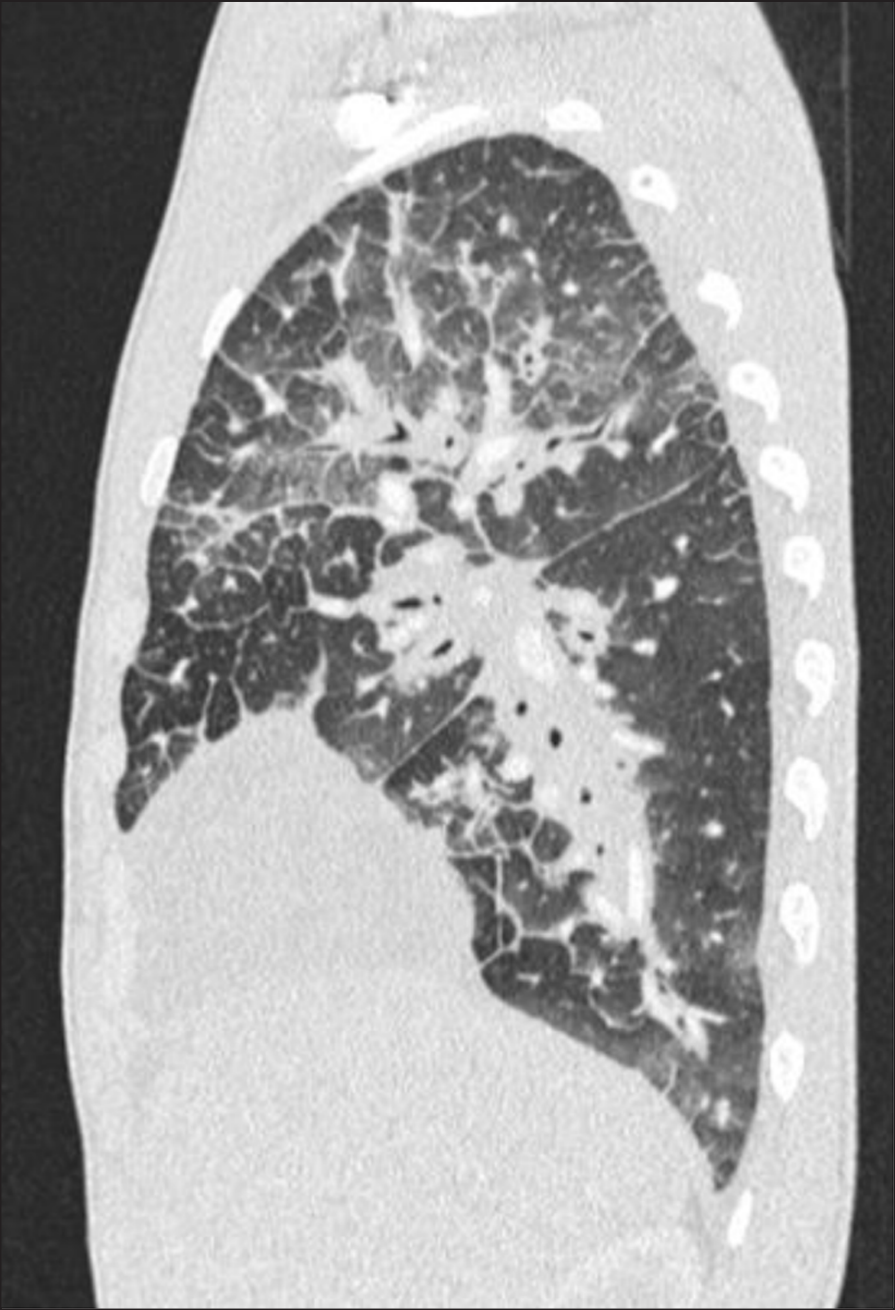


A 20-year-old man complained of exertional dyspnea for two months, cough and asthenia. He had no fever, chest pain or lower limb edema. Past medical history, physical examination and lab results were unremarkable. Cardiac ultrasound showed an abundant compressive pericardial effusion and a “mediastinal mass” that was present on the topographic scan of the chest computed tomography (CT) as a mediastinal widening (Figure 1). The contrast-enhanced chest CT itself comprehensively displayed the pericardial effusion and the diffusely hypodense infiltration of the mediastinum extending to hila (Figure 2). There was diffuse thickening of the lung interstitium and patchy ground glass opacities (Figure 3). A thoracoscopy with pleuro-pericardial window and biopsy was performed and led to the diagnosis of pulmonary lymphangiomatosis. The patient’s symptoms subsided after this surgery followed by multiple pleural punctures. An immunosuppressive treatment with Sirolimus (Rapamicyn) was initiated, and the patient has now had good clinical and radiological evolution for four years.

## Discussion

Diffuse pulmonary lymphangiomatosis is a rare pulmonary lymphatic system disorder characterized by the proliferation and distension of lymphatic vessels. It occurs in children and young adults with equal sex prevalence. It is more aggressive in patients who present at a younger age. Symptoms are nonspecific, including dyspnea, cough and wheezing, hemoptysis, chyloptisis and chest pain. The major symptoms are directly related to pleural and/or pericardial chylous effusions.

The CT findings are quite typical: diffuse interstitial thickening (interlobular, peribronchovascular and pleural), patchy ground glass opacities, pleural thickening, pleural and/or pericardial effusion and mediastinal soft tissue infiltration with no mass effect on mediastinal vessels. Differential diagnosis is limited to other pulmonary lymphatic disorder [1].

Lymphatic imaging is complex, either by invasive technique (lymphangiography associate or not to cross-sectionnal imaging, lymphoscintigraphy) or by non-invasive technique (Magnetic resonance lymphangiography, T2-weighted). Those radiologic exams are not much available except in specialized centers.

Different therapies have been tried like various systemic or chemotherapies, radiological embolization, radiation therapy and surgical approaches including lung transplantation, but with inconstant results. There is no standardized treatment protocol. The prognostic is poor, with progressive dyspnea leading to death from respiratory failure.
